# Thermal imaging of the fetus: An empirical feasibility study

**DOI:** 10.1371/journal.pone.0226755

**Published:** 2020-07-28

**Authors:** Anastasia Topalidou, Garik Markarian, Soo Downe

**Affiliations:** 1 Faculty of Health and Wellbeing, Research in Childbirth and Health Unit, School of Community Health and Midwifery, University of Central Lancashire, Preston, United Kingdom; 2 School of Computing and Communications, Lancaster University, Lancashire, United Kingdom; 3 Rinicare Ltd, United Kingdom; Stony Brook University Health Sciences Center School of Medicine, UNITED STATES

## Abstract

Digital Infrared Thermal Imaging (DITI) has much potential in the field of maternal-fetal health and wellbeing research. The fact that it is totally non-invasive is particularly important in this context. The purpose of this study was, for the first time, to assess DITI’s ability to record fetal presentation and position, and other pregnancy-related physiological factors, via their superficial thermal prints. Ten healthy pregnant women (approximately 34–37 weeks of gestation) were recorded with two thermal imaging cameras (Flir C3 and i3 TE-Q1) from five different viewpoints. Participants’ views about the use of DITI in research and clinical practice were also assessed by a completion of a survey. Free hand polygon region of interests (ROIs) were drawn in order to include the entire anatomical area for investigation. The use of free hand polygon ROIs showed high reliability. Thermal images analysis revealed that fetuses presenting cephalically can be identified by the use of DITI, under specific conditions. Fetal movements influenced the thermal patterns that were produced. Future studies need to verify the heat patterns on the skin related to the placenta location, in order to understand the produced thermal recordings. Pregnant women rated the idea of using DITI in research and clinical practice very highly. This work represents a first contribution towards the use of DITI for the recording of fetal presentation and position. As it does not require direct contact and since it is completely non-invasive, it could be used to record maternal-fetal dynamic dyadic interaction in pregnancy. However, although the preliminary results are promising, further trans-disciplinary studies with a well-established protocol, more sophisticated thermal cameras, and bigger cohorts are needed.

## Introduction

Digital Infrared Thermal Imaging (DITI) or thermography was originally developed for industrial and military applications. Since the early 1960s, medical practitioners began to see the potential of thermal imaging for a range of applications, from the detection of breast cancer, to the investigation of vascular, musculoskeletal and neurological conditions [1–7]. One of the main features that DITI medical applications are based on is that the higher the temperature of a part/area, the more radiation is emitted [8]. A perfect emitter has an emissivity value of 1. The human body with an emissivity of 0.98 is very close to this [7–9]. A thermal image of a human body, or an area of it, is a visual representation of the surface skin temperature. The infrared radiation emitted by the skin surface includes the heat that is transferred from internal structures or events, such as superficial and deep veins, core organs, inflammations, or any conditions with increased blood supply or angiogenesis [4,6,7,10–12]. Depending on thermal sensitivity of the camera, known as Noise Equivalent Temperature Difference (NETD), small temperature differences can be detected in different areas of the body. Through these measurements, thermography has proved its ability to identify ‘hot spots’ related to breast cancer tumors [13] or pathologies in highly vascular deep-lying organs in neonates, like the heart and kidneys [12].

Since DITI is both non-contact and totally non-invasive, it is potentially an optimal tool for use in maternal and neonatal care. However, to date applications in pregnant women have been restricted to the identification of temperature profiles in different parts of the body [4,5,14,15] and placental location [10,11].

Two essential intrauterine structures develop during pregnancy: the placenta and the fetus. Both the placental and fetal temperature are constantly 0.5°C higher than the maternal core temperature [16–19]. Based on the hypothesis *“that the placenta*, *being an arteriovenous “fistula”*, *has a temperature higher that its immediate environment*, *and the heat from this area is transmitted through the adjacent anterior abdominal wall to produce a characteristic pattern of heat emission on its surface*”, in 1966 Millar [11], conducted the first study of placental localisation by thermography with promising results. However, although DITI had been used in veterinary medicine for pregnant animals [20], its ability to detect thermal patterns related to the human fetal location and position and the visualisation of the mother-fetus dyad simultaneously, has been never tested before.

The fetus has a constant temperature difference (ΔΤ) with the maternal core [16–19]. In addition, 84.5% of the heat that is produced by the fetus exits back to the mother through the umbilical circulation, while the remaining 15.5% is dissipated through the fetal skin to the amnion and then through the uterine wall to the maternal abdomen [18]. We used these known parameters to test the DITI’s ability to detect and record the heat that is transferred from the fetus to the maternal abdominal wall.

The purpose of this study was to investigate the feasibility of employing DITI for use in maternity care, and the influence of contextual factors such as environmental and demographic characteristics. The main objective was to investigate if DITI can detect thermal patterns associated with fetal presentation and position, and other pregnancy-related physiological parameters (e.g. placental location). A secondary aim was to identify women’s views about the use of DITI.

## Participants and methods

### Participants

Ten healthy pregnant women (approximately 34–37 weeks of gestation) aged 18–40 years, with no significant medical history, who could read and write in English, took part in the present observational study. Participants were eligible for inclusion if they had a singleton and viable pregnancy with no complications and body mass index (BMI) <30 (before the pregnancy). Women were not eligible for inclusion if they met the following criteria: a) multiple pregnancy, b) previous caesarian section or any abdominal operation, c) dermatological disorders or inflammation in the area of abdomen or lower back, and d) receiving any medication affecting the cardiovascular system or pain medication at the day of exam. All participants were provided with a participant’s information sheet, informing them in detail about the purpose and the protocol of the study. Suitable participants were required to provide written consent. The Ethics and Integrity Committee of University of Central Lancashire, UK, approved this study [STEMH (Science, Technology, Engineering, Medicine and Health) 559].

### Technique and measurement procedure

#### Preparation prior the assessment

As the assessment procedure included the recording of participants’ body surface temperature, prior to any measurement pregnant women were asked to avoid certain activities to ensure the validity of the procedure [7]. Specifically, participants were asked not to:

- undertake any solarium or sunbathing, of the area of the torso (front, side and back view) from the 7^th^ cervical vertebrae (C7) to the 1^st^ sacral vertebrae (S1) in the 5 days before the assessment session,
- apply any ointments or cosmetics to the abdomen and lower back on the day of assessment,
- undertake any waxing, hair plucking and/or shaving on the area of the abdomen 8 hours prior the assessment session,
- apply any hot or cold packs on the abdominal wall or lower back 8 hours before the assessment session,
- perform any physical therapy or exercise 12 hours before the assessment session,
- smoke or drink alcohol at least 12 hours before the measurement,
- wear extremely tight fitting clothing on the area of abdomen and lower back 4 hours before the assessment,
- bathe or shower 1 hour before the assessment session

All measurements were conducted in a closed room with a controlled temperature and relative humidity. Temperature and humidity were continuously recorded from the beginning to the end of the assessment session, with a ThermoPro TP-50 digital monitor (iTronics, USA). Maximum, minimum and average values were noted at the end of the session. Only a member of the research team and the participant (with their companions/partners if required) were in the measurement room, ensuring a consistent number of people in the room (min number of people 2, max number of people 3).

On the arrival at the room, participants were asked to fill a demographic questionnaire (S1 Appendix). Also, women were asked if they were aware of the presentation of their fetus (cephalic presentation, breech presentation or transverse lie). Then participants were asked to remove any jewellery and clothes from the area of assessment (abdomen/middle to lower back). Participants were able to retain clothing below the superior pubic ligament and above the bottom of the bra. Then, they were asked to stand and/or sit for 15 minutes with the abdomen/torso exposed to achieve skin temperature equilibration with the temperature of the room (acclimatisation period) [7]. During the acclimatisation period, participants were able to stand, sit and move freely, as long as they did not have any contact with their abdomen and lower back. The exposed region of interest (ROI) was from the anterior superior iliac spine (ASIS) to the lower ribs.

#### Assessment set up

**Fig 1:**
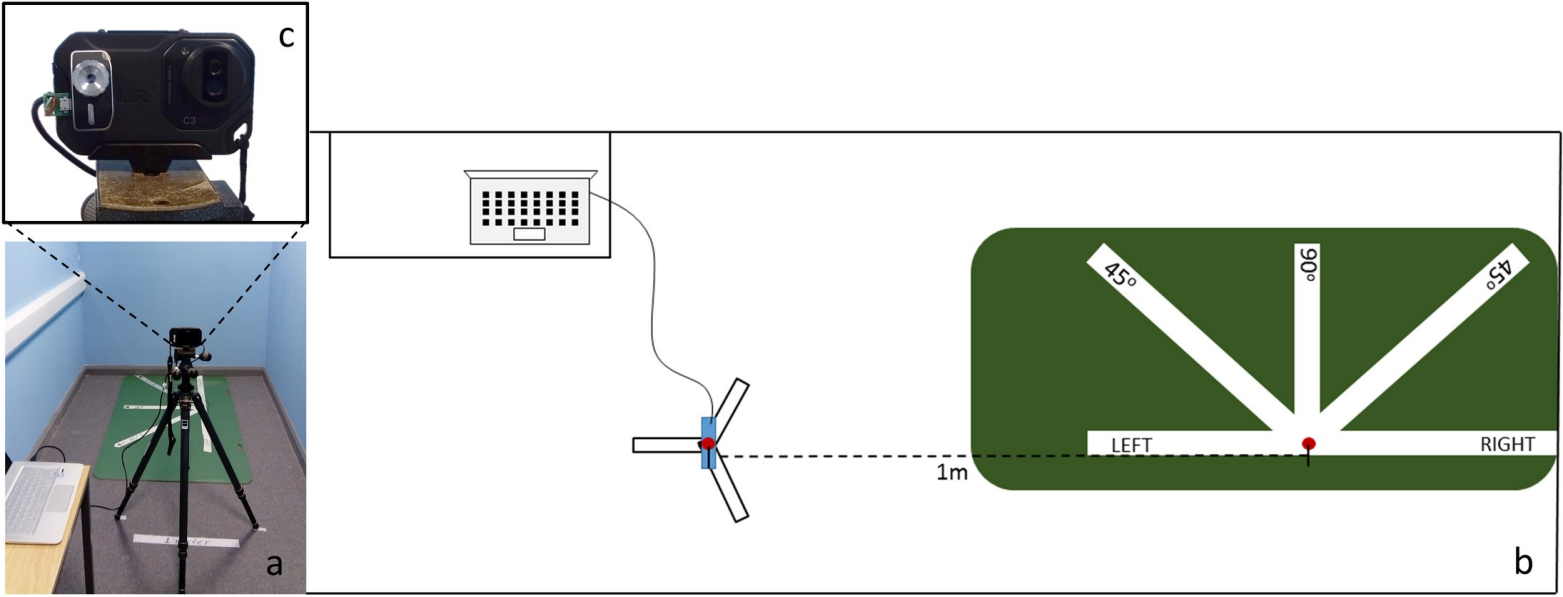
A controlled space free from any objects that could cause heat disturbance was used (a). The participant had to stand in the middle of the mat, with her left side to the cameras, which was placed 1 m away. Every minute the participant had to rotate by 45^o^, stepping on the relevant white line on the mat (b). The lenses from the two cameras were parallel at a distance of 7.5cm from each other (centres of lenses) (c).

For the measurement procedure two thermal imaging cameras (Flir C3 and i3 TE-Q1) and their corresponding software were used (ThermaCam Researcher Professional 2.10 and Thermal Expert Q1 1.5.7). Laptops and other devices were sited away from the participant’s assessment location, to avoid heat disturbance (Fig 1A and 1B). Flir C3 [IR resolution 80X60, Field of View (FOV) 41^o^x31^o^, NETD <100mK] was mounted on a rigid tripod and the i3 TE-V1 (IR resolution 384X288, FOV 56.3^o^x41.8^o^, NETD <50mK) was attached to Flir C3 with a double-sided adhesive tape. The two lenses were parallel and approximately at the same height/level (Fig 1C). The small difference between the height/level of the two lenses was due to the different FOV of the two cameras. The cameras’ lenses were parallel to the area of measurement. The i3 TE-V1 was connected with a customised cable to a laptop (providing power and transfer of the recordings). The height of the tripod was adjusted each time based on the participant’s height, so that the ROI would always be as more as possible in the center of the displayed image/video. The i3 TE-V1 was used for a continuous video recording from the beginning of the measurement (Start point) to the end of the assessment (End point). Both cameras were used to capture two images at every viewpoint (see below). At the end of the session for every participant, 1 thermal video, 10 thermal images from the Filr C3 and 10 thermal images from the i3 TE-V1 were recorded and stored. Both cameras (their lenses) were located 1m away from the spot that participants had to stand (Fig 1B). The room set up is presented in Fig 1. No other equipment, furniture or appliances were in the room of assessment. To achieve electronics equilibrium and assure stable measurement, both cameras were turned on at least 15 minutes before any measurement were taken. An additional manual image calibration (using the shutter operation) was performed on i3 TE-V1, before the beginning of the recording (Start point).

#### Assessment

**Fig 2:**
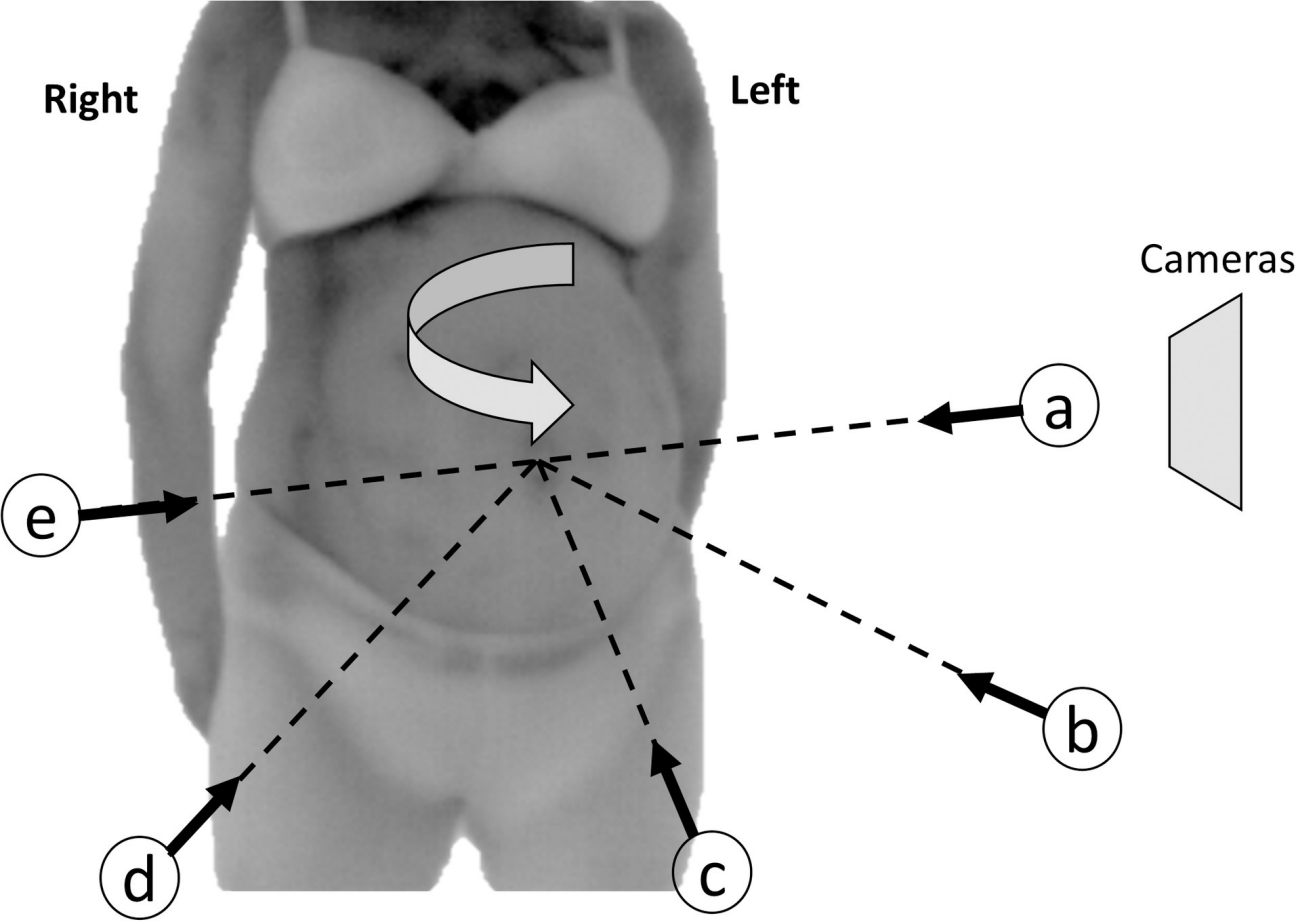
The five viewpoints.

Before the assessment participants were advised on how to stand and turn, following the signs on a specially designed mat (Fig 1). Once participants were confident with the procedure, they were asked to stand still on the central spot on the mat, with both their hands either behind their back or behind their head. At this position, the assessment began with the recording of the left side. Cameras remained stationary, and participants were asked to turn each time by 45^o^ for each viewpoint and remain in this position for 1 minute. The total length of the recording was 5 minutes (1 minute for each viewpoint). The following five viewpoints were recorded: (a) left side view (vertical to left side), (b) 45^o^ degrees left to front view, (c) front view (vertical to the abdomen), (d) 45^o^ degrees right to front view and (e) right side view (vertical to right side) (Fig 2).

At the end of the session, participants were asked if they felt any fetal movements during the procedure from the start point to the end point, and if they could describe the position of the fetus. Finally, participants were asked to complete a survey of their views about the imaging technique (S2 Appendix). The aim of the survey was to investigate participants’ views on the use of thermal imaging for research proposes during pregnancy and/or during antenatal care. The survey consisted of three 5-point Likert-scale questions and four open-ended questions. For the Likert-scale questions the “I don’t know” option was also available, in order to provide the participants with a non-forced choice to respond if they did not wish to offer a specific opinion [21].

### Data management

All responses to demographic questionnaire and participants’ views survey were transferred from the hard copies into Excel files. Room temperature and relative humidity, participants’ perception of fetal position and movements were also reported into an Excel file.

#### Infrared images

The recorded thermograms from both thermal imagers were analysed using the cameras’ corresponding software. Human skin emissivity was set at 0.98 and distance at 1m. Parameters such as atmospheric temperature, reflected temperature and humidity were set for every participant separately based on the recordings from the ThermoPro TP-50. Manual adjustment of level and span was performed (thermal tuning).

#### Qualitative analysis of thermograms

The qualitative description (QD) approach [22] was used for the description of the thermograms that were recorded by both cameras. Thermograms recorded with the i3 TE-V1 were used only for the qualitative analysis, as there was no available option to extract temperature values or perform detailed radiometric analysis. The recorded videos were also non-radiometric. The corresponding software had only one option for ROI selection, which was a square area, without the ability to export temperature values for every pixel. The application of a thermal image segmentation algorithm was no within the scope of the current study.

The analysed images from both cameras were presented to an experienced midwife. The midwife was not aware of the participants’ responses, and was asked to view the images from the five different viewpoints for every participants and report. She was asked if it was possible to identify the fetus, its presentation/position and provide a narrative description. This was compared to participants’ answers about fetal presentation and their perception of fetal position.

#### Quantitative analysis of thermograms

Quantitative analysis was performed only in the images recorded by the FLIR C3. As stated above, for every view point two images were recorded and stored, to ensure the capture of least one good quality image. For the quantitative analysis only one of the two images was selected for the analysis (always the first one captured), if both images were assessed by the researcher as of good quality. Physiological changes in an individual over time (blood flow in superficial veins, fetal movements etc.) affect emitted temperature values. Since the two images, taken for each view point, were separated in time, it was therefore not appropriate to report mean values from the combined data across the two images in each ROI.

**Fig 3:**
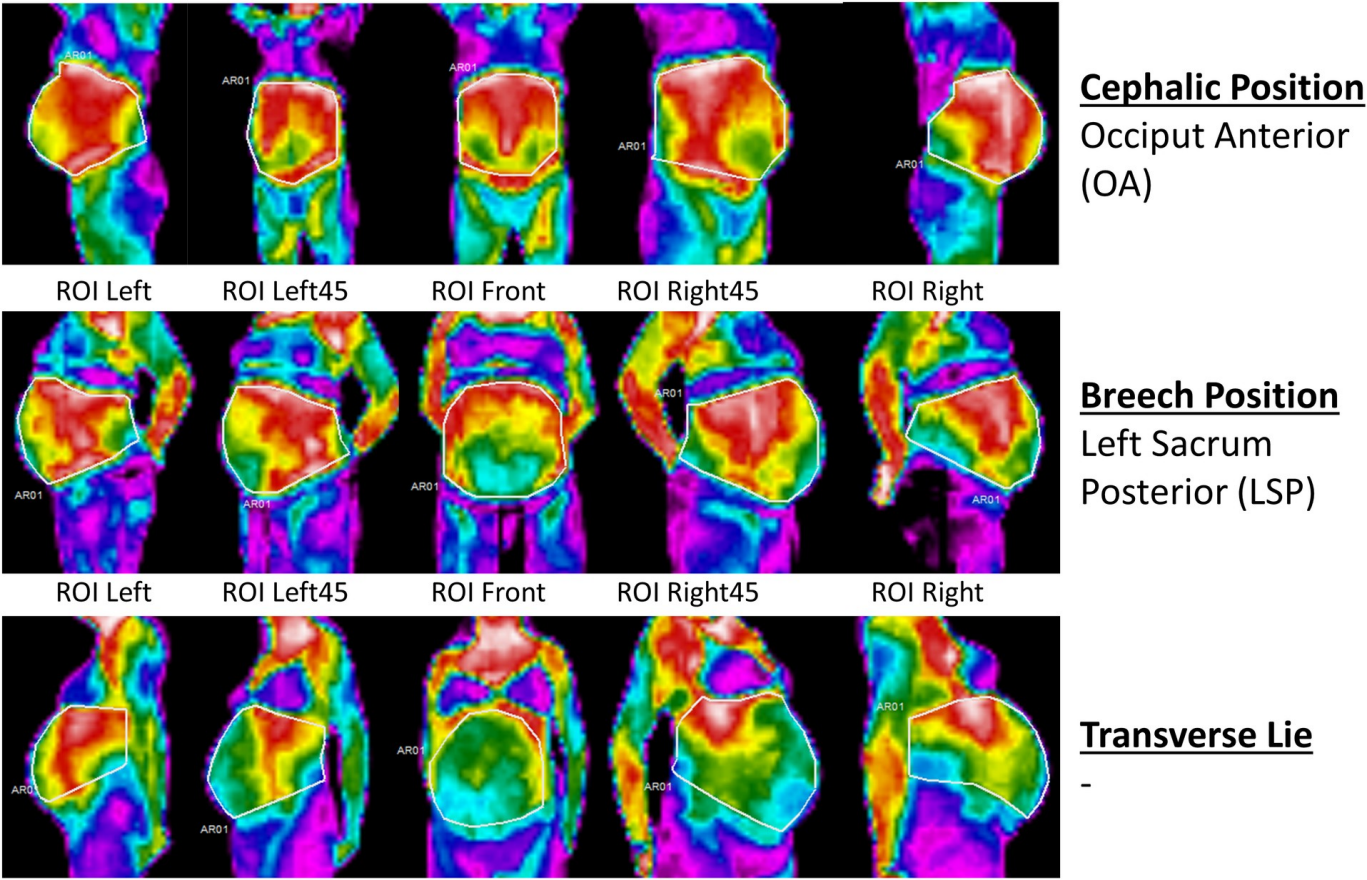
Thermal images from the five viewpoints. Free hand selected polygon ROIs were used for the analysis. Three cases with different fetal positions and no fetal movements during the procedure are presented.

For the analysed images, each ROI was defined using a free hand polygon (Fig 3). The selected ROIs included the exposed abdominal region, excluding any clothing or anatomically irrelevant areas. For every viewpoint one ROI was defined. Therefore, for every participant five ROIs were selected. For every ROI the average (T_avg_), maximum (T_max_) and minimum temperature (T_min_), and the number of pixels were computed. In total for every participant, 20 parameters were counted and analysed. As every participant had different body size and shape, the areas of the selected ROIs could not be equal. For this reason, and in order to keep the size of the analysed region as similar as possible, a minimum pixel count for each ROI in every participant was used. For the left side view (ROI Left) and the 45^o^ degrees left to front view (ROI Left 45), the minimum required pixels were 350. For the front view (ROI Front), the 45^o^ degrees right to front view (ROI Right45) and the right side view (ROI Right), the minimum required pixels were 400. The difference of 50 pixels was based on the distance between the recorded ROI and cameras’ lenses, as during rotation participant’s front and right sides were coming slightly closer to the lenses than the left side. Although the use of a geometrically regular shape (triangle, circle, square) would be able to provide more equal areas, this would not be able to cover the entire region of our interest, without excluding anatomically and physiologically important parts of the body, or including irrelevant data [23].

As the FLIR C3 had a low thermal resolution, and a customised free hand polygonal ROI was used (not a fixed shape with the same area), in order to assure data repeatability, the captured images were analysed twice by the same researcher, with one week interval [24] (intra-rater reliability). The researcher used two different laptops, one for each analyses and ROI selection, and during the second analyses had no access to the previous analysed data and selected ROIs.

### Statistical analysis

SPSS version 24.0 software (IBM Corp. 2016, IBM SPSS Statistics for Windows) was used for all statistical analyses. Descriptive statistics [means and standard deviation (SD)] were used to report the temperature and the relative humidity in the room of the assessment, as well as participants’ parameters (age, height, weight, BMI and weeks of gestation). Frequencies were used to report: the gestational age; how many of the participants had been pregnant before; how many gave birth before and in which presentation and position the fetus was (if this information was known to the participants). Responses to the survey’s questions using the 5-point Likert scale were described as means±SD. Data gathered from open-ended questions were coded and categorised into broad themes.

To ensure the reliability of the analysis, paired t-tests were used to examine differences between the two analyses performed by the same researcher, for every parameter assessed. Intra-rater reliability was determined by calculation of the Intraclass Correlation Coefficient (ICC) (2-way mixed effect model, absolute agreement) with 95% Confidence intervals (CI) [25]. Although a measurement with ICC values greater than 0.6 is considered of good repeatability, in the current study the more strict criteria of Currier [26] was applied: 0.90–0.99 = high reliability, 0.80–0.89 = good reliability, 0.70–0.79 = fair reliability, ≤0.69 = poor reliability. The Standard Error of Measurement (SEM), which has a direct correlation with the reliability of the method, was calculated as SD×√(1 − ICC) [27]. SEM was used for the calculation of the Minimal Detectable Chance (MDC) for 95% degree of confidence. MDC was defined as MDC_95_ = 1.96×SEM×√2 [28].

The impact of the external factors on the recorded temperatures was evaluated. Pearson correlation was used to assess if there was any correlation between the demographics of population and the average room temperature and humidity levels, with the recorded T_avg_, T_max_ and T_min_ in each ROI (for every view point). It was also used to investigate if there was any correlation between the T_avg_, T_max_ and T_min_ in every ROI with the location of the fetus. The strength of the correlation was described using the guide suggested by Evans (1996) [29] for the absolute value of r (0.80≤|r|≤1.00 “very strong”; 0.60≤|r|≤0.79 “strong”; 0.40≤|r|≤0.59 “moderate”; 0.20≤|r|≤0.39 “weak”; 0.00≤|r|≤0.19 “very weak).

All statistical tests were carried at the 5% level of significance.

## Results

During the assessments, the mean room temperature was 21.5°C±1.0°C and the relative humidity 50.4%±2.4% (S3 Appendix–Table 1).

### Participants

The mean (SD) age of the participants was 30.8 (±5.6) years. The mean (SD) height was 1.71 (±0.06) meters and the mean (SD) weight was 83.6 (±11.4) kilograms (S3 Appendix–Table 2). On the day of the assessment, participants’ BMI was 28.5±2.6 kg/m^2^ and their mean (SD) gestational age was 34.6 (±0.6) weeks. Six participants were 35 weeks pregnant, two were 34 weeks, one was 35.5 weeks and one 36 weeks. Five participants had been pregnant before. Four had given birth before. All the participating women knew the presentation of their baby. Eight reported that their baby was in cephalic presentation, one reported that her baby was in breech presentation, and one noted a transverse lie.

### Qualitative analysis of thermograms

Of the n = 10 women, n = 5 who felt no fetal movements during the procedure were confident about the position of their fetus. Of the n = 5 who felt movements during the assessment, only n = 2 were confident to identify the position of their fetus. In the other n = 3 cases the baby was still moving at the end of the procedure, and therefore the position could not be confirmed by the participants. The seven described positions were as followed: n = 2 Cephalic-Right Occiput Anterior (ROA) (no fetal movements), n = 1 Cephalic-Occiput Anterior (OA) (no fetal movements), n = 1 Breech-Left Sacrum Posterior (LSP) (no fetal movements), n = 1 Transverse lie (no fetal movements), n = 1 Cephalic-Left Occiput Posterior (LOP) (fetal movements), n = 1 Cephalic-Left Occiput Transverse (LOT) (fetal movements). The women’s knowledge about the presentation and the position of their fetus was used as ground truth information during the analysis of the images.

### Clinical assessment of the analysed thermal images

The midwife member of the research team provided exactly the same answers for the images presented from both cameras. For this reason, it was not necessary to undertake the planned Bland-Altman plot and analysis, to compare the understanding of the outputs from the two cameras.

**Fig 4:**
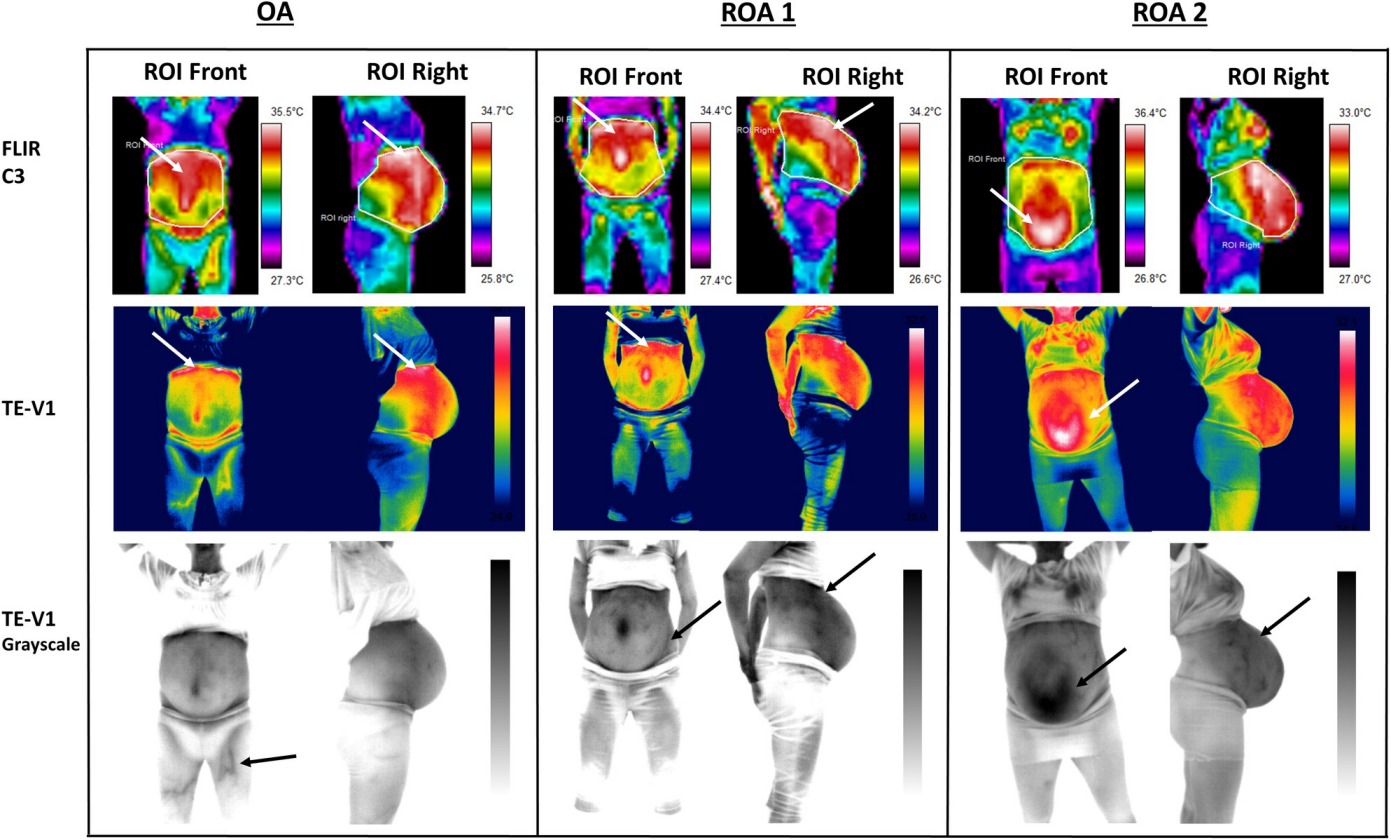
Representative qualitatively analysed thermal images of three women who did not experienced any fetal movements during and/or after the assessment. In all three cases the fetus was closer to the right side, which presented warmer-red regions related to the fetal position/location.

**Fig 5:**
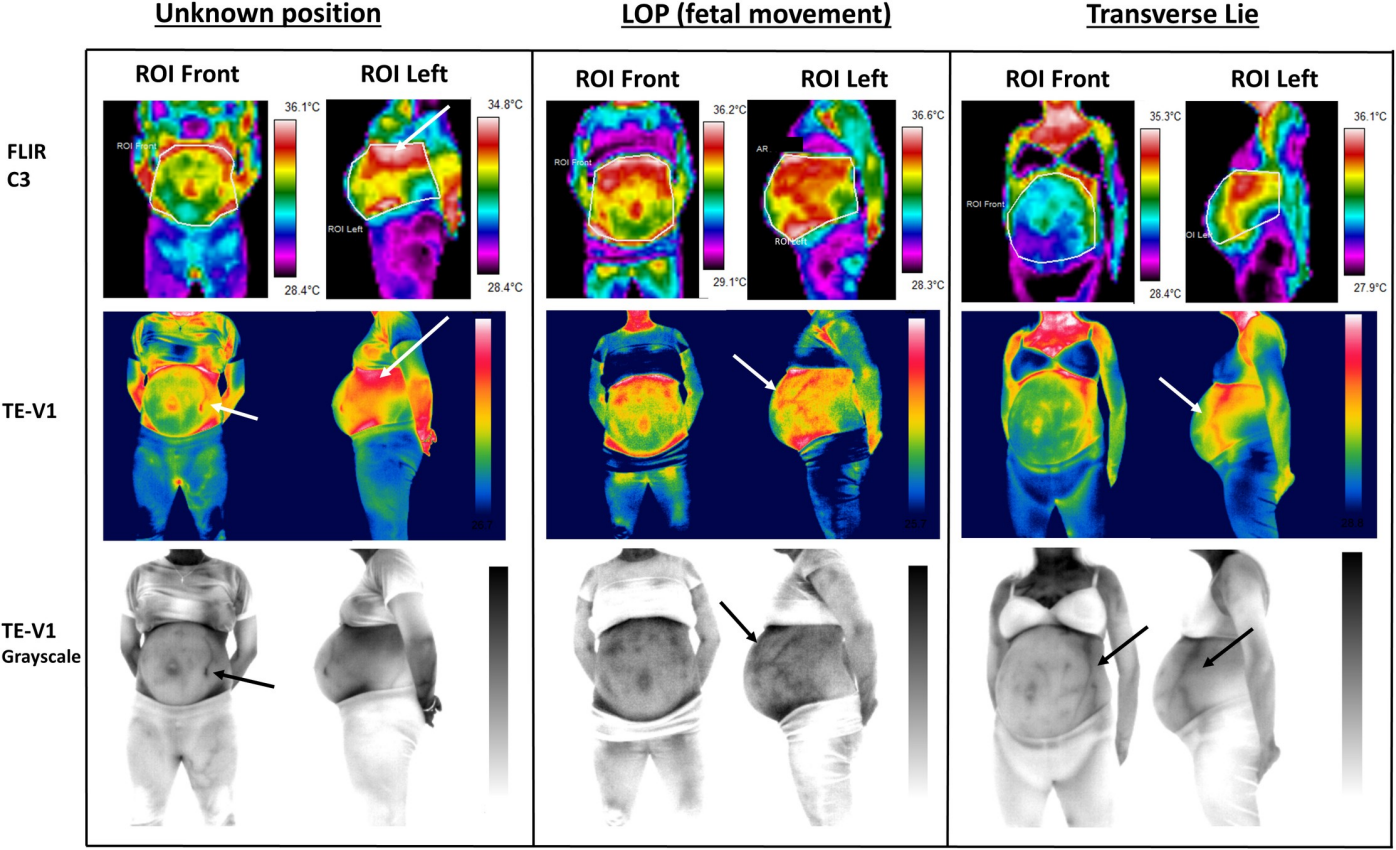
Qualitatively analysed thermal images of: a) One women who experienced fetal movements during and after the procedure, and therefore couldn’t describe or located the position of the fetus (Unknown position), b) One women who experienced fetal movements only during the procedure, and after the end of the procedure could describe the position of her fetus as LOP, and c) One women whose fetus was in transverse lie and did not felt any movements during and/or after the procedure.

There was agreement between the midwife (reading the thermal images) and the woman (perceiving the position and presentation of her fetus), as both noted that the fetus was cephalic and in OA position (Fig 3: Cephalic Position OA and Fig 4: OA). The midwife also identified the fetus as being on the right side for the two positions defined as ROA by the participants, but no further description could be provided (Fig 4: ROA1 and ROA2). For the breech LSP position, the midwife identified the fetus as “*more to the left side*”, but was not able to say if it was breech or cephalic or provide any other information (Fig 3: Breech Position). For these four positions, participants did not experience any movements during or after the procedure. For the remaining six participants the midwife was not able to identify the position of the fetus from the thermal images. For one woman, the fetus was in the transverse lie (Fig 3: Transverse lie and Fig 5: Transverse Lie).

### Pseudocolor analysis

Qualitative pseudocolor analysis (specific colour-temperature response) showed that the absence of fetal movements during and/or after the procedure (Fig 4) gave different thermal patterns compared to the thermograms from the women who experienced fetal activity/movements during and/or after the procedure (Fig 5: Unknown position and LOP). The absence of any internally identified activity/movement resulted in the detection of warmer areas related to the potential fetal position or location of other organs, like the placenta. Specific highly warm areas (white and red colour for FLIR C3 and TE-V1) identified just under the anatomical location of the epigastric region (Fig 4: OA and ROA 1—white arrows), and under the umbilicus in the hypogastric region (Fig 4: ROA 2—white arrows) could be related to the location of the placenta. However, as the identification of placental location was not feasible by another means or technique available in this study, confirmation that the warm areas were related to the placental location was not possible. For the purposes of this study, these areas were described as highly-warm unknown areas (HWUA). For the women who experienced fetal movements, a HWUA was identified only in one case and mainly from the side views (Fig 5: Unknown position–FLIR C3—ROI left—white arrows).

Due to the higher IR resolution and the better thermal sensitivity that the i3 TE-V1 had, some additional physiological characteristics were detected when this camera was used. Specifically, superficial abdominal veins were displayed as warmer red lines (in the colour pallet) (Fig 5: Unknown position, LOP, Transverse lie–TE-V1 –white arrows). In addition to this, grayscale thermograms which provided more contrast, were used to distinguish these veins more easily (Fig 5: TE-V1 grayscale—black arrows). Abdominal surface veins were mostly detectable in women who experienced fetal movements (Fig 5: TE-V1—white arrows, and TE-V1 Grayscale—black arrows), compared to the women who did not (Fig 4: TE-V1 and TE-V1 grayscale). For the women who did not experience any fetal movements abdominal veins were detected mainly in the grayscale only in one case (Fig 4: ROA 2 and ROI Right–TE-V1 grayscale). For the OA case presented in Fig 4, although surface veins were detected on women’s lower limbs, even over her clothing (Fig 4: OA–TE-V1 grayscale—black arrow), no other veins were detected, making the area of interest clear from any interferences.

Another important observation is that women who experienced fetal movements (Fig 5: Unknown position and LOP–ROI Front) had in general cooler ROIs Front, in comparison with the women who did not (Fig 4: OA, ROA 1 and ROA 2 –ROI Front). As shown in Fig 5 (Unknown position and LOP–FLIR C3 and TE-V1) ROIs Front presented basically cold colours (yellow, green, light blue), and were cooler under the epigastric area, in comparison with the same areas of the cases presented in Fig 4 (OA, ROA 1 and ROA2—FLIR C3, and ROI Front—TE-V1).

Although, the woman with the transverse lie (Fig 5) did not experience any fetal movement during or after the procedure, we presented her thermograms with the other two cases (Unknown position and LOP), in which there were no clearly detected warm areas which could be related to the position/location of the fetus. For the transverse lie case, this may be because, a significant part of the fetus is covered by the ilium bones. The only red region illustrated in the ROI Left (FLIR C3 and TE-V1), was related to the presence of a surface abdominal vein, as confirmed by the grayscale thermogram (TE-V1 grayscale–ROI Left). Also, the ROI Front, which was the coolest in comparison with all the other women, was displayed as purple and dark blue in the hypogastic area and as light blue in the epigastric area by the FLIR C3; and as shades of green by the Te-V1 camera. All five view points for the transverse lie case are presented in Fig 3.

Finally, based on the women’s responses about the position of their fetus, the exported thermograms by both FLIR C3 and TE-V1, and the absence of any interfering veins in the ROIs, it was apparent that red areas within each selected ROI Right (Fig 4: OA, ROA 1 and ROA 2), could delineate the position of the fetus.

### Quantitative analysis thermograms

The number of the selected pixels and the computed T_avg_, T_max_, T_min_, for every participant’s five ROIs, as exported during the first and the second analysis performed by the same researcher, are presented in the S4 Appendix and S5 Appendix respectively.

The mean±SD number of pixels that were included in every ROI during the first and the second analysis are shown in Table 1.

**Table 1:** Reliability coefficients for temperature values and number of pixels for each ROI. ICC, SEM and MDC for the two free hand polygon image analyses undertaken using the ThermaCam Researcher Professional 2.10 software.

		REGION OF INTEREST (ROI)				
		LEFT	LEFT 45°	FRONT	RIGHT 45°	RIGHT
Minimum Temperature	**Mean± SD Analysis 1 (°C)**	27.5±2.2	27.3±1.6	29.2±1.4	26.8±1.8	26.9±1.8
	**Mean± SD Analysis 2 (°C)**	27.6±2.0	27.1±1.7	20±1.3	26.9±1.6	26.9±1.6
	**ICC (95% CI)**	0.984 (0.925–0.996)	0.971 (0.887–0.993)	0.967 (0.853–0.992)	0.989 (0.955–0.997)	0.980 (0.918–0.995)
	**SEM (°C)**	0.3	0.3	0.2	0.2	0.2
	**MDC**_**95**_ **(°C)**	0.5	0.6	0.5	0.3	0.5
Maximum Temperature	**Mean± SD Analysis 1 (°C)**	34.1±1.2	34.4±1.4	34.3±1.6	34.4±1.1	33.6±1.0
	**Mean± SD Analysis 2 (°C)**	34.1±1.2	34.3±1.4	34.4±1.5	34.4±1.1	33.6±1.1
	**ICC (95% CI)**	1.000	0.994 (0.978–0.999)	0.994 (0.971–0.999)	1.000	0.996 (0.985–0.999)
	**SEM (°C)**	0.0	0.1	0.1	0.0	0.1
	**MDC**_**95**_ **(°C)**	0.0	0.2	0.2	0.0	0.1
Average Temperature	**Mean± SD Analysis 1 (°C)**	31.8±1.3	32.0±1.3	31.9±1.3	32.0±1.2	31.4±1.1
	**Mean± SD Analysis 2 (°C)**	31.8±1.3	31.9±1.2	31.9±1.2	31.9±1.2	31.4±1.2
	**ICC (95% CI)**	0.999 (0.995–1.000)	0.999 (0.997–1.000)	0.999 (0.995–1.000)	0.999 (0.996–1.000)	0.998 (0.989–1.000)
	**SEM (°C)**	0.0	0.0	0.0	0.1	0.1
	**MDC**_**95**_ **(°C)**	0.1	0.1	0.1	0.1	0.1
Number of Values (pixels)	**Mean± SD Analysis 1**	493.0±55.8	517.5±106.0	591.2±88.7	702.2±92.8	529.5±86.7
	**Mean± SD Analysis 2**	491.1±51.3	532.7±98.8	626.0±113.4	705.7±70.8	542.1±95.2
	**ICC (95% CI)**	0.966 (0.862–0.992)	0.969 (0.880–0.992)	0.900 (0.569–0.976)	0.958 (0.831–0.990)	0.973 (0.896–0.993)
	**SEM**	10	18	32	17	15
	**MDC**_**95**_	19	35	63	33	29

### Intra-rater reliability

There was no statistically significant difference between the first and the second analysis undertaken by AT for all the 20 analysed parameters. The mean values (±SD) of the first and the second analysis, the ICC, the SEM and the MDC_95_, for the exported T_avg_, T_max_ and T_min_ values, including the number of pixels, for all the five ROIs are presented in Table 1. All the parameters showed high reliability displaying ICC>0.90. The SEM for the temperature values ranged from 0.0°C-0.3 ^o^C and the MDC_95_ from 0.0°C-0.6 ^o^C. The SEM for the number of values ranged from 10 to 32 pixels and the MDC_95_ from 19 to 63 pixels.

Based on the above only the values computed during the first analysis used in the following evaluations.

### Bivariate analysis of the temperature values with the environmental and demographic factors

The average room temperature was correlated to the recorded temperatures of some ROIs. Specifically, there was a strong statistically significant correlation with the recorded T_min_ of ROI Left (r = 0.729, p = 0.017), ROI Left45 (r = 0.666, p = 0.036), ROI Right45 (r = 0.657, 0.039), and very strong statistically significant correlation with the ROI Front T_min_ (r = 0.866, p = 0.001). It also showed a moderate correlation, but not statistically significant, with the T_min_ for ROI Right (r = 0.519m p = 0.124) and the T_avg_ for ROI Left45 (r = .0401, p = 0.251).

The average relative humidity presented a negative strong statistically significant correlation with the ROI Left T_max_ (r = -0.738, p = 0.015), the ROI Left45 (r = -0.666, p = 0.036) and the ROI Right (r = -0.669, p = 0.035) T_min_, as well as the T_avg_ of the ROI Left45 (r = -0.657, p = 0.039) and the ROI Right (r = -0.684, p = 0.029). Additionally, the T_min_ for ROI Left and ROI Right45; the T_ma_x for ROI Left, ROI Left45, ROI Front and ROI Right45; and the T_avg_ for ROI Front and ROI Right45 presented negative moderate correlations ranging from r = -0.432 to r = -0.629, which were not statistically significant.

For the demographic parameters, T_min_ of the ROI Front presented strong but not statistically significant correlation with participants’ weight (positive r = 0.626, p = 0.053) and moderate but not statistically significant correlation with participants’ height (positive r = 0.465, p = 0.175), BMI (positive r = 0.548, p = 0.101), previous pregnancy (negative r = -0.516, p = 0.127) and previous birth (negative r = -0.421, p = 0.226). Other parameters that showed non-significant moderate correlation were: T_min_ ROI Left (r = 0.405, p = 0.246) with BMI, T_min_ ROI Right (r = 0.415, p = 0.233) with Height, T_max_ ROI Front (r = -0.563, p = 0.090) and T_avg_ ROI Front (r = -0.489, p = 0.151) with weeks of gestation.

### Bivariate analysis of the temperature values with the fetal location

As there are many positions that a fetus may have, for this calculation the location of the fetus was used based on the maternal description and the qualitative analysis of the images. Specifically for statistical purposes ROA and OA were computed as “1:Right”, LSP, LOP and LOT were computed as “2:Left” and the transverse lie with the three unknown positions were computed as “3:Uknown”.

Contrary to expectations, fetal position showed to have a strong and moderate negative correlation (but not statistically significant) with the T_max_ and T_avg_ of almost all ROIs, which means that the fetus could be located closer to the regions that presented lower maximum and average temperature values.

### Participants’ views survey

All the results are presented in Table 2. Women who participated in the current study graded the idea of using DITI during pregnancy and their overall experience “very high”. They rated some features of thermal imaging, such as being contactless, totally non-invasive, completely safe, and providing real time monitoring as 5/5 (“very good”). All participating women would like to see DITI being used in the future for maternity care. When women were asked ***"****Thinking about all the monitoring you have had in pregnancy*, *how important are the following features for you when your baby is being monitored****"*,** the response “completely safe” was rated most highly, as “very important”, while the “duration of the assessment” had the lowest rating, as “somehow important”. The key issues raised from the open-ended responses are shown in Table 2.

**Table 2:** Participants’ responses to the twenty 5-point Likert scale questions and the reported key issues to the four open-ended questions.

5-point Likert scale		
**Number of responses (n =)**	**Question**	**Mean±SD** *(Very high = 1; High = 2*, *Average = 3*, *Low = 4*, *Very low = 5)*
**10**	How you would rate the idea of using the new imaging technique during pregnancy?	**1.1 ±0.3**
**9**	How you would rate your experience with the whole procedure of the new imaging technique?	**1.0 ±0.0**
**9**	What is your opinion of your images as they were presented to you?	**1.1 ±0.3**
**10**	How comfortable was the procedure for you?	**1.1 ±0.3**
** **	***"Please circle the answer that best describes your inmpression regarding the following features of the new technique"***	**Mean±SD** *(Very poor = 1; Poor = 2*, *Average = 3*, *Good = 4*, *Very good = 5)*
**10**	Duration of assessment (time)	**4.9 ±0.3**
**9**	Quality of images	**4.8 ±0.4**
**10**	Comprehension of images	**4.6 ±0.5**
**10**	Contactless	**5.0 ±0.0**
**10**	Totally non-invasive	**5.0 ±0.0**
**10**	Easy to applied	**4.9 ±0.3**
**10**	Real-time monitoring and recording	**5.0 ±0.0**
**10**	Completely safe	**5.0 ±0.0**
** **	***"Thinking about all the monitoring you have had in pregnancy*, *how important the following features are for you when your baby is being monitored"***	**Mean±SD** *(Very important = 1; Important = 2*, *Neutral = 3*, *Somehow important = 4*, *Not important at all = 5)*
**10**	Duration of assessment (time)	**3.7 ±1.6**
**10**	Quality of images	**2.1 ±1.4**
**10**	Comprehension of images	**2.0 ±1.4**
**10**	Contactless	**3.0 ±1.4**
**10**	Totally non-invasive	**2.8 ±1.7**
**10**	Easy to applied	**2.6 ±1.6**
**10**	Real-time monitoring and recording	**2.3 ±1.3**
**10**	Completely safe	**1.4 ±1.3**
**10**	***"Would you like to see this method being used in the future for pregnant women*?*"***	**"Yes" n = 10 / "No" n = 0**
**Open-ended Questions**		
	Question	Key-Issues
**10**	***"Please explain your answer to the question above"***	"non-invasive"; "safe"; "real-time"; "contactless", "quick, not time-consuming", "monitor fetal movements"
**8**	***"What impressed you most about the new imaging method*?*"***	"completely safe and non-invasive"; "see the images in real-time"; "didn't have to saty still"; "not having anything poking at the abdomen"; "simple to use"
**5**	***What disappointed you most about the new imaging method*?**	"nothing"; "images weren't immediately recognisable"
**3**	***"Please share any additional comments about your experience or suggestions*, *if you have any"***	"excellent experience"; "pleasant experience"; "really interesting"

## Discussion

This preliminary feasibility study has revealed thermal patterns for healthy pregnant women related to the position and location of their fetus and other physiological characteristics.

### Rationale for thermal assessment in the third trimester

The reason for selecting women in the second half of the third trimester was based on the anatomical location of the uterus and its size, and the amniotic fluid volume [30,31] in relation to the expected body volume of the fetus [32]. During the first trimester, the uterus is mainly an intrapelvic organ. It becomes palpatable (and therefore has the potential to be detectable by DITI) only after the 12^th^-14^th^ week [10]. Although thermography could be useful during the second trimester, for the localisation of placenta and differential diagnosis of placenta praevia and low implantation of placenta [10], it is currently challenging to isolate recordings related to the heat that is transferred from the fetus to the maternal abdominal wall at this stage of pregnancy. During the second trimester the amniotic fluid volume shows a weekly increment reaching approximately 800ml by the 28 weeks, while the fetal body volume has a less rapid increase [30–32]. During the third trimester, the volume of the amniotic fluid is much lower in comparison with the volume of the fetal body, which occupies the majority of the space in the uterus. In addition, the abdominal cavity reaches its maximum anterior expanding. This forward expansion of the abdomen in combination with the corresponding arrangement of the abdominal muscles results in a decrease in the thickness of the abdominal tissues, especially on the left and the right side of women’s abdominal area [33]. The combination of these factors facilitates the recording of thermal prints of the heat that is transferred from the intrauterine environment to the surface of the maternal abdominal wall, during the second half of the third trimester. Also, at this stage, the fetal movements are more restricted as there is a limited space within the uterus. In the current study, all the participants were aware of the position of their baby, which was a useful anchor for our observations.

### Influence of environmental and demographic parameters

DITI is highly dependent on the environmental conditions, such as the airflow, the temperature and the relative humidity of the room or place of application [9]. Most previous studies using DITI have been performed in controlled environments with the temperature ranging from 18°C to 25.5°C [4–7,14,15] and the relative humidity between 50%-60% [5,6] The results of the current study showed that the average room temperature had a very strong and strong statistically significant correlation with the minimum recorded temperature (T_min_) of some ROIs. This means that the higher the room temperature the higher the recorded T_min_ values of some areas. However, the maximum (T_max_) and average temperature (T_avg_) showed no statistically significant relation to the room temperature. On the other hand, the relative humidity showed a negative strong statistically significant correlation with the T_max_, T_min_ and T_avg_ of specific ROIs, demonstrating that potentially its role is more important than that of room temperature. This study was the first that revealed the role of the room temperature and humidity in DITI application in pregnant women. To the best of our knowledge there is no study investigating the optimal environmental conditions for DITI application in pregnant woman. Similarly, there are no studies exploring if there is any correlation of the environmental condition with participant’s body temperature. In general, it is known that in order to record good data, the subject of the recording (in this case the pregnant woman) must have a temperature above or below the ambient background temperature. However, the optimal temperature difference (ΔΤ) between the participant and the recorded background remains unknown [9]. Due to the lack of this knowledge most of the researchers try to perform their investigations at environmental temperatures between 23°C-31°C which are the limits of the zone of vasomotor regulation (or the condition of comfort) [34]. Further investigation is needed in order to understand if lower room temperatures, in order to increase the ΔΤ between the participant and the recorded background, and different levels of humidity, could enhance the DITI application during pregnancy.

Apart from that, before any DITI application, an acclimatisation period is required. In a study with pregnant and non-pregnant women, where an hour of thermographic data was recorded, the authors stated that although the usual acclimatisation period is between 15–20 minutes, temperature variations were continued over this period of time [15]. As shown for the first time in this study, fetal movements can have a direct effect in the recorded thermal patterns. Therefore, these continuous temperature variations, reported by Falzon et al 2018 [15], may be related to internal adaptations due to fetal movements, which altered the patterns of the transferred heat to the maternal abdomen and therefore the superficial thermal prints. This might be particularly relevant, as in the study concerned, pregnant women at their 28–30 weeks of gestation took part [15]. The number of movements, their frequency and strength increase and reach their maximum between 28–32 weeks [35]. Also, as the anterior abdominal wall has superior, inferior and lateral vascular supply [36], vasodilation response can dynamically influence the recorded emission from the abdominal wall of both non-pregnant and pregnant women [34] during the acclimatisation period. In a study conducted in 1952 it was found that some areas of the body (such as the fingers) do not show a consistent pattern even after 90min of testing [34]. This means that each area of the body could require a different acclimatisation period. As the investigation of the use of DITI in pregnancy is a relatively new field, a 3-dimensional map of the maternal abdomen in correlation with the required acclimatisation period, under a range of controlled and real-life conditions, is an important area of primary research.

Finally, the potential influence of demographic characteristics requires further testing with larger samples, and including more variation between women. For example, increased subcutaneous abdominal adipose tissue in obese adults creates a substantial insulating layer that blunts abdominal heat transfer [37]. A study which investigated if the skin temperature of pregnant women was influenced by their age, BMI and gestational age identified that age and the BMI had direct, positive and negative respectively, correlation with the skin temperature. Although the location of the placenta and the fetus, as internal structures of higher temperature were not taken into consideration, the authors highlighted the need for future work comparing pregnant and non-pregnant women [14].

### Qualitative and quantitative analysis of thermograms

This was the first study where women were asked about the position and presentation of their fetus and their perception of fetal movements, providing important ground truth information.

The results of the current study showed that the thermal images were more informative when the fetus was still during the assessment. A relatively constant fetal position resulted in a more “smooth”, laminar transition of the heat via the amniotic fluid to the maternal abdomen. This stable environment lead to more distinguishable thermal prints on the surface of the abdomen. Fetal movements created turbulent motions with known effects (eddy diffusivity, higher thermal transfer rates etc.). Therefore, fetal movements play an important role in the produced thermal prints. Potentially, advanced analytics of continuous high frequency recordings will be able to identify dynamic thermal changes over time and correlate them with the presence of fetal movements. Further transdisciplinary studies are needed to establish this in future.

### Identifying fetal presentation/position and the role of placental location

This study suggests that a fetus with a cephalic presentation produces a more recognisable outline of their presentation/position, at least within the capacity of the cameras used for this study. Anterior positions were also more easily identifiable (Fig 3: OA-ROI Right and Fig 4: OA, ROA 1 and ROA 2 –ROI Right for both FLIR C3 and TE_VI1). For the only case in breech position, although a warmer pattern was recorded from the left side (Fig 3: Breech Position-ROI Left and ROI Left45), the fetus’s LSP position in combination with the potential presence of anterior wall placenta, made it difficult to recognise if the fetus was in cephalic or breech position and its exact orientation. The recording of HWUA without prior information on what they might be, made the understanding of some cases difficult.

Since the mid-60’s, thermography has been widely used as a research tool for placental localisation. The findings from the early black and white thermal recordings were confirmed either by a subsequent caesarean section (in cases of placental praevia) or by x-ray. Results suggested that placental location, pelvic inflammations or abdominal masses could be identified [10,11]. As the placenta and the fetus have exactly the same temperature [16], it is expected that they will produce similar thermal patterns of heat emission on the abdominal surface. In addition, the placenta can be in front of the fetus when a recording is made from a specific angle. Based on the above placental location is a valuable component of fetal thermal imaging.

In the current study, for the one case where the fetus was in transverse lie, no obviously identifiable warm areas that could be related to the fetal position were recorded. In addition, the ROI Front presented the coolest recordings in comparison with the other cases. The absence of a determined warm area in combination with the unexpectedly cold recording of the front view point could be an indication of a transverse lie, however more transverse lie cases are needed to define this preliminary finding.

### Viewing angles

In this study, lateral views (ROI Left and ROI Right, as well as ROI Left45 and ROI Right45) were the optimal viewpoints for the thermal images obtained. The majority of the studies to date recorded their images either from the front viewpoint only or front and back (lower back) [4,5,10,11,15]. Anterior viewpoints may be influenced by the expansion of the linea alba (connective tissue) and the distribution of subcutaneous adipose tissue in some women. Only two studies in pregnant women used a lateral viewpoint. One assessed the mean skin temperature of the selected ROIs before and after two types of physical exercises [38] and the other the thermal symmetry between the two sides by selecting a small circular ROI, excluding a big part of the abdominal area [14]. However, none of the above studies took into consideration the potential location of the fetus and the placenta.

Based on the abovementioned results related to the recordings from the different viewpoints, primary experimental studies under controlled conditions to establish norms for thermal imaging in pregnant women should, ideally, involve a system with five synchronised cameras, one for every viewpoint, with continuous high-frequency video recording.

Another parameter that has to be taken into consideration is superficial abdominal veins. The increased blood supply makes them easily identifiable thermally. A method to exclude them before any further advance analytics would be beneficial for future projects in the area of DITI application in pregnancy.

### ROI segmentation and analysis

The free hand selected polygon ROIs showed high reliability and seem to be superior to shape-specific ROIs (which are more often used in thermal imaging of the human body in other medical applications), so this could be the optimal method of analysis for thermal imaging data in pregnancy.

Usually, during the analysis of a ROI the T_avg_, T_max_ and T_min_ values are calculated. Although, these values can provide important information, they cannot be really used when the comparison of an area is needed. For DITI studies in humans a single value analysis (regression to mean) hides important information related to the physiology and anatomy of the area or region of investigation. The assessment of the recorded areas as 2D matrices is part of authors’ next stage of analysis.

### Participants’ views

To the best of our knowledge this is the first study where participants’ have been asked about their views on the use of thermal imaging, for research and clinical practice. The main advantages of DITI (non-contact, non-invasive, easy to use) were of value to the participants, who agreed that DITI could be a useful clinical tool in the future.

### Limitations

Both cameras used in the current study had some limitations. While FLIR C3 has the ability to provide radiometric data, it has low thermal resolution and thermal sensitivity. On the other hand, TE-Q1 has higher thermal resolution and sensitivity, but the extraction of radiometric data was not possible. The current study was performed with low-cost mobile thermal cameras, providing information on whether accessible and affordable detectors can be used for DITI applications in maternity care. Despite the promising preliminary results from our study and the recent reports from other researchers that these types of the cameras have sufficient resolution for studies performed in controlled environments [39], especially when imaging techniques like thermal voxel, thermal gradient flow and optimal quantisation are applied [40]; we have to highlight the importance of the NETD and the number of pixels especially when small differences have to be detected. In conditions where the fetal position and/or placental location are detected via their superficial thermal print, while the vascular zones have to be clearly distinguished, a more advanced camera with the ability to record dynamic radiometric data could be beneficial.

Another limitation of this study is the relatively small sample size. Although the participants were relatively homogenous, it was difficult to define thermally the many different fetal positions with such a small number of participants.

Finally, maternal perception of the presentation/position of their fetus was used in the current study as background information. Although the determination and confirmation of the fetal and placental position by any other means would be ideal, this is not possible due to several factors. The use of a diagnostic ultrasound would be difficult before the DITI application, as its two bio-effects, heating and cavitation would affect the results. The tissue warming results from the acoustic energy deposited in the tissues by ultrasound absorption [41,42]. Studies in models or animals detected an elevation of fetal or model temperature up to 5°C. Apart from the thermally adverse bio-effects, it remains unknown how much time is needed for the fetus and the tissues to return to their physiological temperature and how this affects the heat transfer mechanism [43]. In addition, the perfect removal of the gel from the skin could be another challenge. On the other hand, although ultrasound could be used after the DITI application to confirm the location of the placenta, it would be quite difficult to determine the position of the fetus, as it is known that ultrasound waves stimulate the fetus, resulting in an increase of 90% in fetal movements [44]. Abdominal palpation could be an alternative, though this could involve heat transfer from health professional’s or midwife’s hands, and thermal energy from friction, if the palpation would be applied before the DIGI recording. However, given that abdominal palpation has only moderate accuracy to determine fetal position [45], future studies in this area could consider combining maternal perception and abdominal palpation (after the DITI recording) to improve accuracy.

The current study is the first study ever to present thermal images of the fetus. Within the limited technical capacities of the thermal cameras used and the issues that were identified by this study and are described above, we have shown that cephalic presentation and some fetal positions can be identified. We have also shown that the lateral viewpoints are the most effective, and that women are very positive about the potential use of the technique. Future studies with more precise detectors need to be undertaken, to determine optimal conditions for DITI applications during pregnancy and obtain all the benefits that thermography can add in the area of maternal/fetal health research.

### Recommendations for further research

The human body temperature is an indicator of health. Many health or disease conditions can be identified through information about temperature alterations and/or distribution. DITI provides the ability to record and illustrate a thermal map of the emitted radiation by the skin surface [1]. As it is a completely non-invasive method, that does not require any contact and can be used at distance with the participant being mobile, it can have many potential applications in the area of maternity services.

Firstly, thermal imaging can be helpful for conditions that are related to temperature alterations, for either the mother or the fetus or both of them.

Also, it is known that the mother and fetus is an interrelated system, a dyad [46,47]. Many health- and/or wellbeing-related functions and conditions do not originate in or affect only one “part” of the system. Therefore, the answers for many scientific questions in this area rely on understanding this dynamic dyadic system. To the best of our knowledge, the mother and fetus have never been investigated as a dyad in dynamic motion. As shown from the results of the current study, DITI is able to record the mother and her patterns, by capturing the naturally emitted radiation, while displaying the heat that is transferred from highly vascular internal organs and the fetus to the surface of the skin. The latest development in thermal detectors which offered the ability for continuous video recording with ≥30Hz frame rate and thermal sensitivity of <15mK in some cooled cameras [1,4], could support future applications enabling the investigation and understanding of maternal-fetal dyad in motion, for instance, during labour.

In addition, DITI enables the detection of the competing subdivisions of the autonomic nervous system, with high reliability and unlike conventional methods provides the multidimensional ability to record perspiration [48], cutaneous and subcutaneous temperature variations and patterns [49,50], breathing rate [2,51], blood flow [52], cardiac pulse [2] (as pulsative blood flow modulates tissue temperature), and many more, in a non-contact way. As pregnancy is a particular situation, during which a woman undergoes many normal physiological changes, DITI could enable their observation, especially in case when a pathology that requires a non-contact and non-invasive method for both the mother and the fetus, arises.

## Conclusion

Undertaking DIGI recording in pregnant women is a promising task, but there are challenges to overcome, as several factors and variables have to be taken into consideration.

The detection and the location of the placenta and the fetus with the use of DITI is possible under specific conditions. The current study showed promising results related to the location of fetuses in cephalic position, revealing that the lateral view abdominal recordings produce more informative results than anterior view recordings. In addition, the presence or the absence of fetal movements during the DITI application plays an important role in relation to the thermal patterns of the abdomen.

A pregnant woman with the fetus is a dynamic dyadic interrelated system that should be investigated as such, without excluding their interdependent influences. DITI has many potential applications in the field of maternity care, mainly due to its non-invasive and non-contact properties. The preliminary results from this study show that DITI has potential for use in the investigation and detection of physiological conditions and alterations related to pregnancy and to fetal factors. Additional studies with cameras with higher thermal resolution and NETD, and advanced analytics of dynamic thermographic data are required, in order to identify thermal patterns related to all the potential fetal positions, and to fetal movement.

## Supporting information

S1 Appendix Participants’ demographic information questionnaire. (PDF)

S2 Appendix Survey of women’s views about the use of thermal imaging in pregnancy. (PDF)

S3 Appendix The average temperature and humidity values of the assessment room and participants’ demographic characteristics. (PDF)

S4 Appendix The minimum, maximum, average temperature values from the selected ROIs and the number of pixels in each ROI, as exported during the 1^st^ analysis and ROI selection. (PDF)

S5 Appendix The minimum, maximum, average temperature values from the selected ROIs and the number of pixels in each ROI, as exported during the 2^nd^ analysis and ROI selection. (PDF)
