# Supplementary material for: Thermal imaging of the fetus: An empirical feasibility study
Source: PLoS One. 2020 Jul 28;15(7):e0226755. doi: 10.1371/journal.pone.0226755 (PMC7386602; doi:10.1371/journal.pone.0226755)
Supplement: S1 Appendix — (PDF) [file pone.0226755.s001.pdf]

Demographic Information Questionnaire

PILOT STUDY TO INVESTIGATE THE USE OF THERMAL IMAGING FOR RESEARCH AND CLINICAL USE IN PREGNANCY

Protocol/Study number:_____________ Participant identification number: ____________

1. Gender: Female ☑ Male ☐
2. Age: ________________(years) Would rather not say ☐
3. Height: ________________(cm) Would rather not say ☐
4. Weight: ________________(Kg) Would rather not say ☐
5. Can you please tell us how many weeks pregnant you are? ________(weeks)
6. Have you ever been pregnant before? Yes ☐ No ☐ Would rather not say ☐
7. Have you ever given birth before? Yes ☐ No ☐ Would rather not say ☐
