# Supplementary material for: Thermal imaging of the fetus: An empirical feasibility study
Source: PLoS One. 2020 Jul 28;15(7):e0226755. doi: 10.1371/journal.pone.0226755 (PMC7386602; doi:10.1371/journal.pone.0226755)
Supplement: S2 Appendix — (PDF) [file pone.0226755.s002.pdf]

Survey of women's views about the use of thermal imaging in pregnancy

Protocol/Study number:______________ Participant identification number: _____________

You have kindly agreed to take part in our study of the use of thermal imaging in pregnancy. We now invite you to tell us about your views and experiences of this new process. Thank you for taking part in this final, important part of our study. The survey only takes about 5 minutes.

This survey is **anonymous**. This means that your name can never be linked to your answers. Please, do not write your name on the survey.

Please circle the answer that best describes your experience/opinion:

	Very high	High	Average	Low	Very low	I don't know
**How you would rate the idea of using the new imaging technique during pregnancy?**	1	2	3	4	5	-
**How you would rate your experience with the whole procedure of the new imaging technique?**	1	2	3	4	5	-
**What is your opinion of your images as they were presented to you?**	1	2	3	4	5	-
**How comfortable was the procedure for you?**	1	2	3	4	5	-

Please circle the answer that best describes <u>your impression</u> regarding the following features of the new technique:

	Very poor	Poor	Average	Good	Very good	I don't know
Duration of assessment (time)	1	2	3	4	5	-
Quality of images	1	2	3	4	5	-
Comprehension of images	1	2	3	4	5	-
Contactless	1	2	3	4	5	-
Totally non-invasive	1	2	3	4	5	-
Easy to applied	1	2	3	4	5	-
Real-time monitoring and recording	1	2	3	4	5	-
Completely safe	1	2	3	4	5	-

Thinking about all the monitoring you have had in pregnancy, please circle the answer that best describes <u>how important</u> the following features are for you when your baby is being monitored:

	Very important	Important	Neutral	Somehow important	Not important at all	I don't know
Duration of assessment (time)	1	2	3	4	5	-
Quality of images	1	2	3	4	5	-
Comprehension of images	1	2	3	4	5	-
Contactless	1	2	3	4	5	-
Totally non-invasive	1	2	3	4	5	-
Easy to applied	1	2	3	4	5	-
Real-time monitoring and recording	1	2	3	4	5	-
Completely safe	1	2	3	4	5	-

Would you like to see this method being used in the future for pregnant women?

Yes ☐

No ☐

Please explain your answer to the question above:

____

____

____

What impressed you most about the new imaging method?

____

____

____

What disappointed you most about the new imaging method?

____

____

____

Please share any additional comments about your experience or suggestions, if you have any.

____

____

____

Thank you for your time!

Please hand the form back to the researcher before you leave
