# Supplementary material for: Thermal imaging of the fetus: An empirical feasibility study
Source: PLoS One. 2020 Jul 28;15(7):e0226755. doi: 10.1371/journal.pone.0226755 (PMC7386602; doi:10.1371/journal.pone.0226755)
Supplement: S3 Appendix — (PDF) [file pone.0226755.s003.pdf]

Table 1. The average temperature (degree Celsius) and the average level of humidity (%) of the room, during each assessment.

N_Participants	Avg_Temp °C	Avg_Hum %
1	22.4	48.00
2	20.9	51.00
3	22.0	49.50
4	22.7	47.50
5	21.4	50.00
6	22.4	51.50
7	21.8	49.00
8	21.9	55.00
9	20.1	53.50
10	19.7	48.50

Table 2. Participants age (in years), height (m), weight (kg) and BMI (kg/m^2)

N_participants	Age	Height	Weight	BMI
1	28	1.63	73.50	27.66
2	30	1.70	73.70	25.50
3	39	1.73	93.20	31.32
4	34	1.80	99.60	30.62
5	39	1.65	77.30	28.39
6	25	1.72	93.40	31.57
7	27	1.71	82.60	28.25
8	33	1.82	97.20	29.34
9	22	1.65	78.30	28.94
10	31	1.70	67.00	23.18
