# Supplementary material for: Thermal imaging of the fetus: An empirical feasibility study
Source: PLoS One. 2020 Jul 28;15(7):e0226755. doi: 10.1371/journal.pone.0226755 (PMC7386602; doi:10.1371/journal.pone.0226755)
Supplement: S5 Appendix — (PDF) [file pone.0226755.s005.pdf]

S2 Appendix. The minimum, maximum, average temperature values from the selected ROIs and the number of pixels in each ROI, as exported during the 2nd analysis and ROI selection.

2nd analysis and free hand polygonic ROI selection

ROI Left

P	T_{min}	T_{max}	T_{max}-T_{min}	T_{avg}	T SD	Pixels
1	28.7	33.8	5.1	32.4	0.9	486
2	27.9	35.5	7.6	32.5	1.2	517
3	27.6	34.4	6.8	32.6	1.0	492
4	29.9	33.4	3.5	31.7	0.6	539
5	28.8	34.2	5.5	31.7	1.1	386
6	29.7	34.3	4.6	32.3	1.0	440
7	28.1	36.1	8.0	33.5	1.1	580
8	24.3	32.8	8.5	29.7	1.6	529
9	24.0	31.9	7.9	29.3	1.3	450
10	24.5	34.3	9.8	32.0	1.7	511

ROI Right

P	T_{min}	T_{max}	T_{max}-T_{min}	T_{avg}	T SD	Pixels
1	27.2	33.0	5.8	31.4	1.1	447.0
2	27.1	34.7	7.6	32.2	1.4	511.0
3	28.1	33.7	5.6	32.0	1.1	546.0
4	30.7	33.6	2.8	32.3	0.6	428.0
5	24.6	33.7	9.0	30.1	1.4	630.0
6	26.6	34.1	7.5	32.0	1.2	492.0
7	27.9	35.1	7.2	32.8	1.1	698.0
8	25.2	32.4	7.2	29.9	1.5	593.0
9	25.0	31.9	6.9	29.7	1.1	474.0
10	26.4	34.0	7.6	32.0	1.2	476.0

ROI Left45

P	T_{min}	T_{max}	T_{max}-T_{min}	T_{avg}	T SD	Pixels
1	28.5	35.7	7.2	32.9	1.0	492
2	27.6	35.5	7.9	32.7	0.9	369
3	29.3	34.5	5.2	32.8	0.8	548
4	28.7	33.6	5.0	31.8	0.8	775
5	27.2	34.1	6.9	31.2	1.2	517
6	28.3	35.2	6.9	32.5	1.0	424
7	28.3	36.6	8.3	33.7	1.1	538
8	24.8	32.9	8.1	30.5	1.4	532
9	24.7	31.9	7.3	29.4	1.2	478
10	25.9	34.1	8.2	32.0	1.2	502

ROI Front

P	T_{min}	T_{max}	T_{max}-T_{min}	T_{avg}	T SD	Pixels
1	30.0	36.4	6.4	31.4	1.2	669
2	27.5	35.2	7.7	32.2	0.9	497
3	29.9	33.3	3.4	32.0	0.8	668
4	30.1	33.8	3.7	32.3	0.7	764
5	29.2	33.4	4.2	30.1	0.7	601
6	30.3	34.5	4.2	32.0	0.7	495
7	30.4	36.2	5.8	32.8	0.8	611
8	30.3	34.3	4.1	29.9	0.6	547
9	26.6	31.0	4.4	29.7	0.6	551
10	28.0	34.4	6.4	32.0	0.8	509

ROI Right45

P	T_{min}	T_{max}	T_{max}-T_{min}	T_{avg}	T SD	Pixels
1	27.4	35.2	7.8	32.4	0.9	614.0
2	28.1	35.2	7.1	33.0	1.0	739.0
3	27.0	34.1	7.1	31.9	1.1	685.0
4	28.0	34.3	6.3	32.7	0.8	741.0
5	27.6	33.8	6.2	30.3	1.1	782.0
6	29.0	34.9	5.9	32.5	1.0	607.0
7	27.9	36.3	8.4	33.5	1.1	870.0
8	24.8	33.3	8.5	30.7	1.2	723.0
9	23.6	32.2	8.6	29.9	1.1	709.0
10	25.0	34.7	9.7	32.6	1.2	552.0

P: Participants; T_{min}: Minimum Temperature; T_{max}: Maximum Temperature; T_{max}-T_{min}: Maximum - Minimum Temperature; T_{avg}: Avegare Temperature; T SD: Standard Deviation for Temperature; Pixels: Number of Pixels analysed.

Temperature values are presented in degrees Celsius.
